# An Unusual Cause of Oxalate Nephropathy: Acute Kidney Injury After Ingestion of Dried Bilimbi Fruit

**DOI:** 10.7759/cureus.109671

**Published:** 2026-05-26

**Authors:** Sophia L Hoff, Ariana Gaspert, Luzia Nigg Calanca

**Affiliations:** 1 Department of Internal Medicine, Kantonsspital Winterthur, Winterthur, CHE; 2 Department of Pathology and Molecular Pathology, University Hospital of Zürich, Zürich, CHE; 3 Department of Nephrology, Kantonsspital Winterthur, Winterthur, CHE

**Keywords:** acute kidney injury, averrhoa bilimbi, bilimbi fruit, hyperoxaluria, oxalate nephropathy

## Abstract

This case report describes a 56-year-old patient who developed acute kidney injury (AKI) requiring temporary dialysis following oral ingestion of dried *Averrhoa bilimbi* (or bilimbi fruit). The pathophysiology is attributed to the high oxalate content of the fruit, which can precipitate as calcium oxalate crystals within the renal tubules and lead to tubular obstruction, inflammation, and acute tubular injury. While previous cases have reported AKI after the consumption of fresh bilimbi fruit or its juice, this is, to our knowledge, the first documented case following the ingestion of dried bilimbi fruit. We describe the pathophysiology of secondary hyperoxaluria and its diagnostic evaluation, focusing on a thorough review of medications, comorbidities, detailed dietary history, and nutritional habits. Although the presence of oxalate crystals in a urine sample can help in the diagnostic workup, their absence does not exclude oxalate nephropathy, and renal biopsy remains the gold standard for confirmation. Treatment is supportive, focusing on the cessation of oxalate intake, adequate hydration, and, if necessary, temporary renal replacement therapy. The sooner the above-mentioned measures are taken, the more favorable the prognosis. However, chronic kidney disease (CKD) may persist.

This case report highlights the need for heightened clinical suspicion and comprehensive dietary and medical assessment in patients presenting with unexplained AKI.

## Introduction

The Southeast Asian bilimbi fruit (*Averrhoa bilimbi*) belongs to the family Oxalidaceae and is commonly known as cucumber tree. It is used in its countries of origin as a natural remedy for arterial hypertension, dyslipidemia, and diabetes mellitus [1]. It is also a widely used ingredient in South Indian cuisine [2]. The fruit contains exceptionally high levels of oxalate, also referred to as oxalic acid [3], and can cause acute kidney injury (AKI) due to its high oxalic acid content and the resulting renal deposition of calcium oxalate, leading to secondary oxalate nephropathy. In addition to increased oxalate ingestion, secondary hyperoxaluria can result from increased intake of oxalate precursors, increased intestinal absorption of oxalate, or impaired oxalate handling, for example, due to fat malabsorption [4,5]. In contrast to secondary forms, primary hyperoxaluria is caused by rare genetic defects in hepatic metabolism.

To our knowledge, this case report describes the first reported instance of AKI associated with the ingestion of dried bilimbi fruit. The drying process removes water, so it is reasonable to assume that the oxalate concentration of the fruit increases per unit weight and that even consuming a small amount of dried fruit can lead to a high oxalic acid load. This high oxalate intake is particularly dangerous for patients with pre-existing chronic kidney disease (CKD) or risk factors for it, such as hypertension and diabetes mellitus, as this population may use *Averrhoa bilimbi* as a natural remedy for these conditions.

We emphasize that natural remedies, as well as commonly used food ingredients such as bilimbi fruit, can cause significant secondary oxalate nephropathy. These factors should therefore be considered during history-taking in cases of unexplained AKI or when oxalate nephropathy is identified on kidney biopsy without a clear etiology.

## Case presentation

A 56-year-old male patient from the Philippines presented to the ED of a nearby hospital with nausea, vomiting, and diarrhea, which had started four days earlier following the ingestion of several dried, uncooked bilimbi fruits. Two days after the onset of symptoms, he experienced severe abdominal pain, causing him to call an ambulance. The patient stated that he habitually used small amounts of dried bilimbi fruit to spice his food, but because of pronounced hunger, he ate several dried fruits at once on an empty stomach. The patient's medical history included arterial hypertension, treated with candesartan/hydrochlorothiazide 16/12.5 mg twice daily, bisoprolol 2.5 mg daily, and lercanidipine 10 mg daily. He continued the antihypertensive and diuretic therapy despite diarrhea, vomiting, and limited fluid intake. Travel and environmental history were unremarkable, as was his sexual history. The patient worked as a nurse and reported no illicit drug use. Family history was notable for renal failure of unclear etiology requiring dialysis in an aunt at the age of 50 years.

On admission, the patient presented with hypertension of up to 196/102 mmHg, which was treated several times with IV urapidil. His heart rate was normal. He was afebrile and had normal oxygen saturation on room air. On clinical examination at the external hospital, slight peripheral edema of the lower extremities was noted, as well as abdominal guarding, most pronounced in the right upper quadrant. Laboratory analysis showed an elevated creatinine level of 869 µmol/L (reference range: 64-111 µmol/L). The last creatinine assessment by his general practitioner four years earlier had shown a creatinine level of 93 µmol/L, corresponding to a glomerular filtration rate of 87 mL/min/1.73 m² (Chronic Kidney Disease Epidemiology Collaboration (CKD-EPI) formula; reference value: >90 mL/min/1.73 m²) [6]. No previous urine findings were available. There was no record of urine production before his admission; however, the patient described declining urine output in the days leading up to his hospitalization. Therefore, we assumed AKI stage 3 according to the Kidney Disease: Improving Global Outcomes (KDIGO) guidelines [7] upon hospital admission. The laboratory results at presentation also showed increased urea (28 mmol/L; reference range: 3.0-9.2 mmol/L), hyperuricemia (676 µmol/L; reference range: 210-420 µmol/L), hyperphosphatemia (2.12 mmol/L; reference range: 0.74-1.52 mmol/L), and elevated inflammatory markers, including leukocytes of 14.9 G/L and CRP of 51 mg/L (reference range for leukocytes: 3.0-9.6 G/L; reference value for CRP: <5 mg/L). Venous blood gas analysis showed metabolic acidosis, with a pH of 7.29 and a bicarbonate level of 16.9 mmol/L (reference range: 22-29 mmol/L); potassium was normal at 4.0 mmol/L. Urine sediment analysis from the referring hospital reported 0-4 leukocytes (reference value: <5) and 5-20 RBCs (reference value: <3) per high-power field. Our hospital's urine analysis showed mixed glomerular and tubular proteinuria with marked hematuria without acanthocytes, possibly due to a previously failed attempt to place a urinary catheter (Table 1). The mixed glomerular and tubular proteinuria suggested acute tubular and glomerular damage, the latter possibly resulting from severe hypertension. No oxalate crystals were detected in the urine.

**Table 1:** Urine sediment on admission to our hospital

Parameter	Reference values	Patient values
Leukocytes	<20/µL	62/µL
Erythrocytes	<20/µL	>900/µL
Hyaline casts	Negative	Negative
Pathologic casts	Negative	Negative
Squamous epithelial cells	Negative	Negative
Transitional epithelial cells	Negative	+
Bacteria	Negative	Negative
Crystals	Negative	Negative
Acanthocytes	<5%	0%
Protein-to-creatinine ratio	<300 mg/g	936 mg/g
Albumin-to-creatinine ratio	<30 mg/g	488 mg/g

Due to the severely reduced kidney function with no ultrasound evidence of a postrenal cause, the patient was transferred to our hospital. Doppler sonography ruled out renal infarction and renal vein thrombosis but showed increased peripheral resistance indices of 0.8 in the area of the interlobular arteries in both kidneys, which, together with the hyperechogenic parenchyma, was consistent with AKI. Rapidly progressive glomerulonephritis, myeloma cast nephropathy, or infectious causes of AKI were considered. However, antinuclear antibodies (ANA), antineutrophil cytoplasmic antibodies (ANCA), anti-glomerular basement membrane antibodies (anti-GBM), hepatitis C screening, and HIV testing were negative; complement factors C3 and C4, immunofixation, and protein electrophoresis were normal. Positive anti-HBs and anti-HBc antibodies, together with negative HBs antigen and undetectable hepatitis B DNA, indicated a resolved hepatitis B infection. There was no history of recent infection. In combination with normal complement factors C3 and C4, postinfectious glomerulonephritis was considered unlikely. Furthermore, there were no clinical signs of infection, such as dysuria, flank pain, fever, hypotension, or tachycardia, and no infectious focus was identified on the abdominal ultrasound described above. The mild elevation of inflammatory parameters, including leukocytes and CRP, was monitored and spontaneously normalized over time; therefore, the inflammation was attributed to uremia. Lactate dehydrogenase (LDH) was slightly elevated at 363 U/L (reference value: <220 U/L), but there was no anemia or hyperbilirubinemia suggesting hemolysis, and the platelet count was normal at 258 G/L (reference range: 150-400 G/L). Therefore, severe thrombotic microangiopathy was an unlikely cause of the AKI, despite the high blood pressure readings. Schistocytes on the peripheral blood smear were not reported, but no explicit search was conducted for them. A local form of thrombotic microangiopathy limited to the kidneys could not initially be excluded; however, there was no evidence of it in the kidney biopsy performed later. Acute interstitial nephritis was considered unlikely, as the patient had not recently taken any new medications, in particular, no nonsteroidal anti-inflammatory drugs or antibiotics.

**Figure 1:**
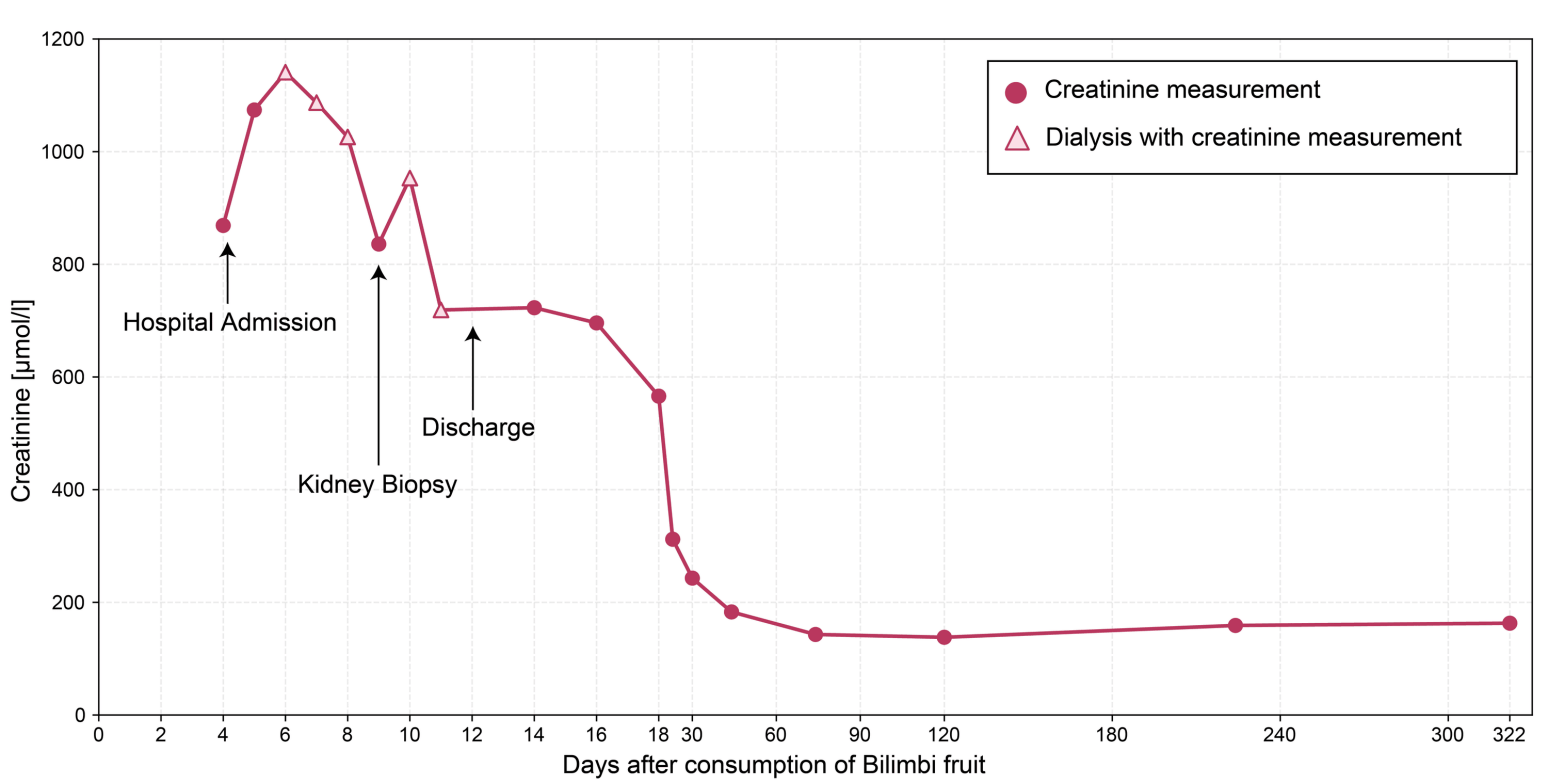
Serum creatinine levels over time following dried bilimbi fruit ingestion.

At first, a prerenal component of the AKI was considered in the context of GI fluid loss, including diarrhea and vomiting for two days, and low fluid intake with continued diuretic and antihypertensive therapy, including hydrochlorothiazide, candesartan, bisoprolol, and lercanidipine. Despite the high blood pressure in the context of stress and abdominal pain, we could not rule out volume depletion, especially since no further peripheral edema was observed upon admission to our hospital. Therefore, the potentially nephrotoxic antihypertensive medications candesartan and hydrochlorothiazide were withheld. Hypertension was initially managed with intravenous urapidil, and the doses of lercanidipine and bisoprolol were doubled. Simultaneously, cautious intravenous fluid replacement was initiated with one liter of isotonic, plasma-adapted electrolyte solution (Ringerfundin) over 24 hours, followed by 1.5 liters over the next 24 hours. Oral fluid intake was approximately 2.5 liters per day. With a further increase in creatinine up to 1141 µmol/L, accompanied by a weight gain of 3 kg, oliguria with 400 mL of urine output during the first two days of hospitalization, persistent hypertension with blood pressure values up to 177/103 mmHg, and abdominal pain suggestive of uremic gastropathy, hemodialysis was started on the third day of hospitalization, corresponding to the sixth day after ingestion of the bilimbi fruit (Figure 1 and Table 2). Antihypertensive therapy was intensified with doxazosin 4 mg, and diuretic therapy with torasemide was started at 10 mg daily. During dialysis and after increasing the torasemide dose to 50 mg daily, urine output gradually improved, reaching 1750 mL/24 h five days after admission. A kidney biopsy was performed on the fifth day of hospitalization and nine days after ingestion of the bilimbi fruit.

**Table 2:** Timeline of clinical events after dried bilimbi fruit ingestion.

Days after consumption of dried bilimbi fruit	Event
0	Ingestion of several dried bilimbi fruits on an empty stomach
0-4	Onset of nausea, vomiting, and diarrhea; antihypertensive and diuretic therapy continued
4	Severe abdominal pain prompting presentation to the emergency department of another hospital; acute kidney injury (AKI) stage 3 of unclear etiology, with creatinine of 869 µmol/L
4	Transfer to our hospital; candesartan and hydrochlorothiazide withheld, antihypertensive therapy intensified, and cautious intravenous fluid resuscitation initiated
5	Persistent oliguria and weight gain of 3 kg
6	Initiation of intermittent hemodialysis; serum creatinine peaked at 1141 µmol/L
9	Kidney biopsy showing oxalate nephropathy with acute tubular injury
12	Discharge after five hemodialysis sessions
322	Last outpatient follow-up at an external hospital, with serum creatinine of 163 µmol/L

**Figure 2:**
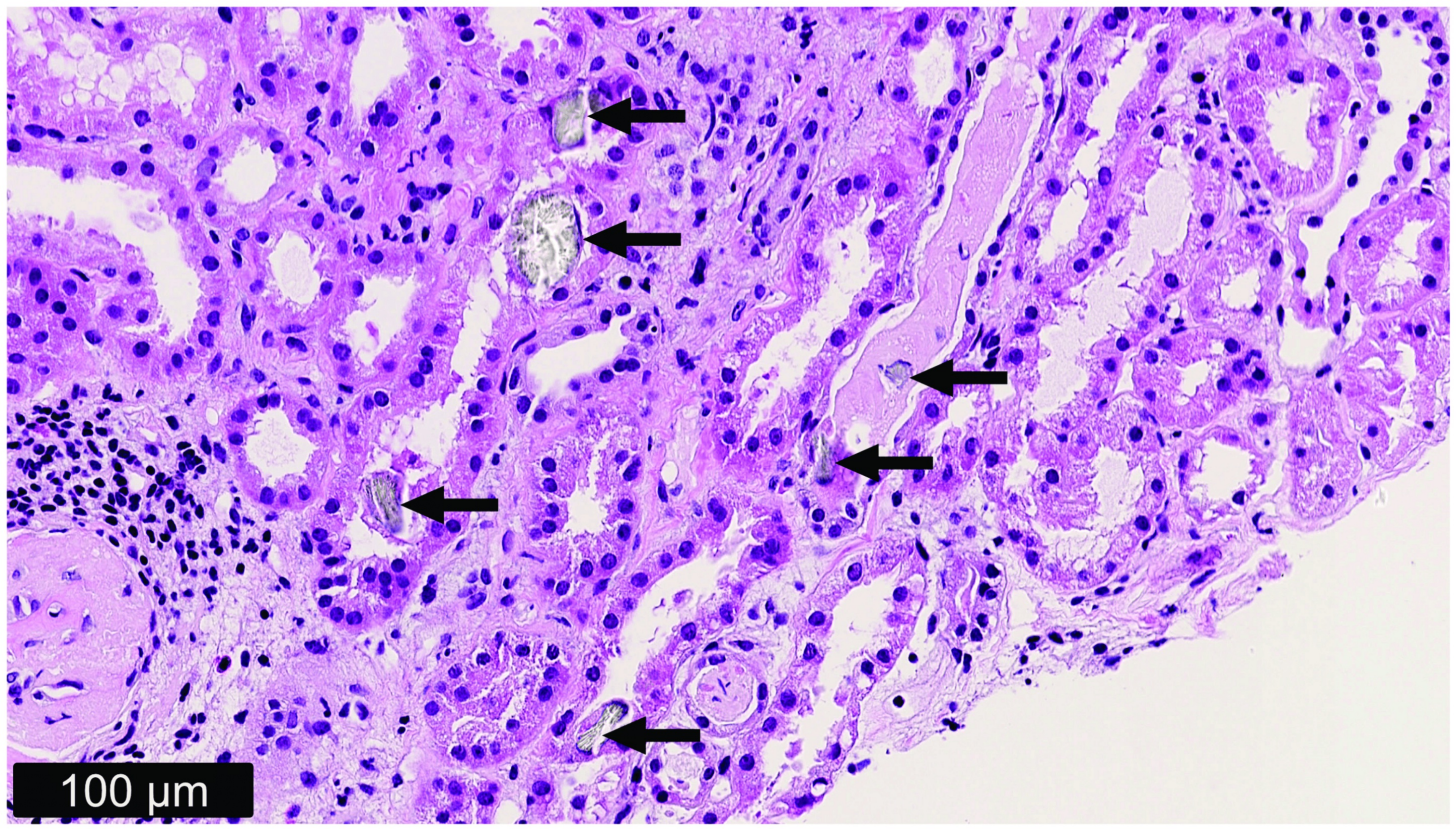
Kidney biopsy showing renal tubules with intraluminal birefringent crystals under polarized light, consistent with calcium oxalate crystals (arrows; H&E stain).

Kidney biopsy showed acute tubular injury with birefringent crystals, consistent with oxalate crystals, which we considered to be the cause of the AKI and oxalate nephropathy (Figure 2). There were numerous extravasations of Tamm-Horsfall protein, some with accompanying lymphohistiocytic inflammation and increased numbers of eosinophilic granulocytes. These findings suggest obstruction, possibly originally caused by the oxalate crystals, as the biopsy was not performed until nine days after ingestion of the bilimbi fruits. Apart from this accompanying inflammation, there was no diffuse interstitial inflammation, no granulomas, and no tubulitis; therefore, there was no evidence of distinct tubulointerstitial nephritis. Additional findings included moderate to severe arteriolar hyalinosis and arteries with moderate intimal fibrosis and elastosis, consistent with changes due to known arterial hypertension. In summary, taking the clinical context into account, the oxalate nephropathy was most likely caused by the large amount of oxalate ingested through dried bilimbi fruit, leading to acute tubular damage due to the deposition of calcium oxalate crystals. A concomitant prerenal component of AKI is possible due to vomiting and diarrhea, exacerbated by the intake of a renin-angiotensin-aldosterone system blocker and a diuretic. The patient also appears to have had pre-existing chronic hypertensive kidney disease.

After five hemodialysis sessions, the patient's uremic abdominal pain improved considerably, and the creatinine values before the dialysis sessions decreased. Therefore, dialysis was halted, and the patient was discharged. During the following outpatient nephrology consultations, both the creatinine levels and the patient's uremic symptoms further improved. The last creatinine value, 322 days after bilimbi consumption, was 163 µmol/L (Figure 1 and Table 2).

## Discussion

Oxalate nephropathy is caused by calcium oxalate crystal deposition in the renal tubules, leading to an acute and/or chronic decline in kidney function. Plasma oxalate originates from normal hepatic metabolism (60-80%) and intestinal absorption (20-40%) [4]. Since oxalate is excreted via glomerular filtration and tubular secretion [4], excess oxalate can cause kidney damage. Oxalate nephropathy can be primary or secondary. Primary forms are autosomal recessive diseases caused by variants in three genes involved in hepatic glyoxylate metabolism and may lead to CKD, eventually requiring renal replacement therapy. Since this case report discusses a form of secondary hyperoxaluria with AKI, we will focus on this subgroup and provide a short overview of the mechanisms.

Secondary hyperoxaluria is categorized according to its etiology (Table 3). General treatment principles, which are partly independent of etiology, are summarized in a separate table (Table 4). Enteric hyperoxaluria usually occurs in the context of fat malabsorption and steatorrhea, for example, in short bowel syndrome, after bariatric surgery, in exocrine pancreatic insufficiency, or as a result of orlistat therapy. In these cases, calcium in the intestine is bound by free fatty acids and is no longer available for binding and fecal excretion of oxalate, thereby increasing oxalate absorption and its deposition in the renal tubules. In our case, however, a different form of secondary hyperoxaluria was present, caused by increased oral intake of highly oxalate-rich food. This form of hyperoxaluria can also be caused by the intake of oxalic acid precursors, such as high amounts of ascorbic acid (vitamin C) or ethylene glycol (antifreeze) [8].

**Table 3:** Subtypes and mechanisms of secondary hyperoxaluria. Data are summarized from references [4,5].

Type of secondary hyperoxaluria	Mechanism
Enteric hyperoxaluria	Fat malabsorption, such as in Crohn’s disease, short bowel syndrome, bariatric surgery, exocrine pancreatic insufficiency, or orlistat therapy
	Free fatty acids bind calcium in the intestine
	Less calcium is available to bind oxalate, resulting in increased intestinal oxalate absorption
Increased oral intake of oxalate or oxalate precursors	Oxalate-rich foods, vitamin C, ethylene glycol, methoxyflurane, and naftidrofuryl oxalate
	Increased systemic oxalate load, resulting in increased renal oxalate excretion and/or deposition
Reduced oxalate degradation by intestinal bacteria	Antibiotic intake, with reduced intestinal oxalate degradation

**Table 4:** General treatment principles for secondary hyperoxaluria. Data are summarized from references [4,5].

Treatment of secondary hyperoxaluria
Treatment of the underlying cause, if possible, e.g., pancreatic enzyme replacement
High fluid intake, aiming for 2-3 L of urine output per day
Dietary changes: lower oxalate and fat intake, with adequate calcium intake
Oral calcium supplements, preferably taken with meals, to bind oxalate in the intestine
Inhibition of calcium oxalate crystallization by alkalinizing the urine with potassium citrate
Oral bacteria/enzyme-based therapies to enhance enteric oxalate degradation, e.g., *Oxalobacter formigenes* or oxalate decarboxylase/reloxaliase; these remain investigational or not routinely established for secondary hyperoxaluria

It is important to note that the prognosis of enteric hyperoxaluria caused by an underlying condition, such as short bowel syndrome, is considerably poorer than that of acute dietary hyperoxaluria, as described in our case. This is because the underlying mechanism, namely increased enteric oxalate absorption, often cannot be fully corrected. Early recognition of secondary hyperoxaluria is important, particularly in high-risk populations, such as post-bariatric surgery patients or patients with inflammatory bowel disease, especially in the presence of malabsorption or bowel resection, because untreated oxalate nephropathy can lead to CKD, eventually requiring renal replacement therapy.

The bilimbi fruit described in our case has a significantly higher oxalate content than most other fruits, with levels reported up to 1032 mg/100 g [3]. Most published case reports of AKI due to *Averrhoa bilimbi* relate to the consumption of raw fruit or juice [3]. However, because drying removes water content, the dried form of bilimbi fruit is expected to contain a higher oxalate concentration per gram than the fresh fruit. Although a direct quantitative comparison with the specific batch consumed by our patient was not possible, this mechanism is likely to result in a markedly increased oxalate load, even with small ingested amounts. Soil conditions and season also play a significant role in the oxalate content of *Averrhoa bilimbi*. For example, the highest oxalate levels have been reported in semi-ripe fruits during the rainy season [3]. Another risk factor for developing oxalate nephropathy following the ingestion of oxalate-rich foods is consumption on an empty stomach, as in our patient and supported by other case reports [2]. Calcium and magnesium in other foods can bind intestinal oxalate and promote its fecal excretion, thereby reducing oxalate absorption [9]. Another exotic fruit with high oxalate content is star fruit, for which there are more case reports of oxalate nephropathy. Depending on how it is prepared, the oxalate content ranges from 54 mg/100 mL in conventionally produced canned juice to 820 mg/100 mL in freshly pressed juice [10]. Other common foods rich in oxalate include Swiss chard, spinach, nuts, rhubarb, black tea, and dark chocolate (Table 5).

**Table 5:** Approximate dietary oxalate content. Data are summarized from references [1,3,4,11].

Food/beverage	Approximate oxalate content
Swiss chard	650 mg/100 g
Spinach	442-571 mg/100 g
Rhubarb	460-573 mg/100 g
Sweet potatoes	280-570 mg/100 g
Nuts	200-550 mg/100 g
Cocoa powder	385-470 mg/100 g
Beetroot	72-181 mg/100 g
Dark chocolate	11-98 mg/100 g
Iced tea/black tea, brewed	23-30 mg/100 mL
Bilimbi fruit	25.1 mg/100 g in one reported sample; up to 1032 mg/100 g reported in half-ripe/rainy-season fruit

Oxalic acid can both promote the formation of calcium oxalate kidney stones and exert a direct toxic effect on kidney tubules and the interstitium through the formation of oxalate crystals that are endocytosed by tubular epithelial cells. This can cause tubulointerstitial inflammation and even fibrosis, as well as tubular obstruction due to calcium oxalate crystals. Other postulated mechanisms of nephrotoxicity include direct oxidative stress, which leads to cytotoxic effects and apoptosis, as well as inflammation caused by the crystals and impairment of intracellular calcium homeostasis [9]. There are a few case reports of AKI after consuming bilimbi fruit [1,2,3,9,12]. In these cases, however, it was consumed as juice or in large quantities as fresh fruit; in contrast, the patient in our case ingested several bilimbi fruits in dried form, which ultimately led to AKI necessitating temporary renal replacement therapy. The consumption of bilimbi fruit may be underreported, as it is commonly used in South and Southeast Asian cuisines, including in dried form. Urinary oxalate crystals cannot always be found, as in our case, especially in patients with established kidney injury at presentation. Occasionally, increased 24-hour urinary oxalate excretion of up to 60 mg/24 h has been described [1]. In our patient, no 24-hour urinary oxalate excretion test was performed.

There are a few limitations to bear in mind regarding our case report. First, the exact amount of dried bilimbi fruit ingested could not be determined retrospectively, as the patient was unable to recall the precise quantity beyond describing it as “several dried fruits.” This limits any dose-response interpretation. Second, fluid-balance data were incomplete: input and output records from the external hospital and ambulance transport were unavailable, and even approximate trends could not be reconstructed for the pre-admission period. The records kept during the hospital stay were not entirely accurate, as the patient did not reliably report all his oral fluid intake. However, the estimated positive fluid balance was confirmed by the 3 kg weight gain during the first two days of hospitalization. Although the clinical distinction between prerenal and intrinsic kidney injury was based in part on assumptions, kidney biopsy confirmed that oxalate nephropathy was a major contributing factor to the AKI. Third, neither serum nor 24-hour urinary oxalate levels were measured. While the diagnosis was ultimately confirmed histologically, direct biochemical quantification of the oxalate burden would have strengthened the causal attribution. Fourth, although thrombotic microangiopathy was considered clinically unlikely and was not supported by the kidney biopsy, no formal evaluation for schistocytes on peripheral blood smear was performed, and thrombotic microangiopathy therefore could not be excluded with full certainty on laboratory grounds alone. These limitations reflect the real-world conditions under which the case was managed and should be considered when interpreting the diagnostic reasoning presented.

Our case report is consistent with previously published case reports regarding the course and duration of kidney disease. Patients initially present with AKI, often requiring dialysis at least temporarily; calcium oxalate crystals with acute tubular injury are visible on kidney biopsy, and serum creatinine levels usually improve within 2-6 weeks. However, as in our patient, creatinine levels do not always return fully to baseline, and CKD may persist.

## Conclusions

In this case report, the ingestion of dried bilimbi fruit and the resulting oxalate burden were the most likely major triggers for acute kidney injury, with additional prerenal and hemodynamic contributors, leading to a temporary need for dialysis. The extent of the kidney injury aligns with that previously described in other case reports following the consumption of fresh bilimbi fruit or bilimbi juice. Since dried bilimbi fruit is commonly used both as a seasoning and as a natural remedy for hypertension or diabetes, careful history-taking is essential to assess oxalate intake as a potential risk factor for kidney injury. Several factors likely contributed to the severity of the kidney damage in this case, including the consumption of dried bilimbi fruit on an empty stomach and the prerenal component caused by GI fluid losses and continued antihypertensive and diuretic therapy in an outpatient setting, all occurring against the background of presumed chronic kidney disease due to hypertension. Secondary oxalate nephropathy due to high oxalate intake generally has a more favorable prognosis than other secondary oxalate nephropathies and does not always require dialysis. Nevertheless, significant kidney damage may persist despite correction of the elevated oxalate intake. In contrast, oxalate nephropathy in the context of short bowel syndrome often has a poorer prognosis, particularly when intestinal oxalate absorption cannot be significantly reduced.

Although kidney injury due to the ingestion of *Averrhoa bilimbi* is rare, we emphasize the importance of obtaining a thorough medical history in patients presenting with acute kidney injury. Particularly in cases with unexplained oxalate deposition on kidney biopsy, patients should be questioned not only about the intake of exotic fruits but also about dietary habits and the use of natural remedies. Thus, in addition to eliciting the medical and medication history, environmental history, travel history, nutritional history, and socioeconomic context may provide information vital to establishing the correct diagnosis.
